# Unilateral Pulmonary Edema Associated With Prolonged Lateral Decubitus Positioning During Tocolytic Therapy in Pregnancy: A Case Report

**DOI:** 10.7759/cureus.103398

**Published:** 2026-02-11

**Authors:** Kei Takumi, Toshihito Mihara, Mao Kinoshita

**Affiliations:** 1 Department of Anesthesiology, Kyoto Prefectural University of Medicine, Kyoto, JPN; 2 Department of Anesthesiology, Japanese Red Cross Kyoto Daiichi Hospital, Kyoto, JPN

**Keywords:** lateral decubitus position, pregnancy, ritodrine, tocolytic therapy, unilateral pulmonary edema

## Abstract

Peripartum pulmonary edema is rare but potentially life-threatening for both the mother and fetus. Unilateral pulmonary edema is particularly uncommon and can mimic pneumonia, delaying appropriate diagnosis because of atypical radiologic findings. A 28-year-old pregnant woman (G3P1) hospitalized for threatened preterm labor received ritodrine hydrochloride and magnesium sulfate. At gestational week 25, she suddenly developed chest pain, dyspnea, and hypoxemia. Chest X-ray revealed unilateral, left-sided pulmonary infiltrates. Cardiac evaluation showed no evidence of cardiogenic pulmonary edema, and infection was excluded, suggesting drug-induced pulmonary edema. Tocolytic agents were discontinued; however, respiratory failure necessitated emergency cesarean delivery. Postoperatively, pulmonary edema rapidly improved with intensive care. A detailed history revealed prolonged left lateral decubitus positioning before symptom onset. Drug-induced pulmonary edema combined with prolonged lateral positioning may predispose pregnant women to unilateral pulmonary edema. Awareness of positional effects during tocolytic therapy is important for early diagnosis.

## Introduction

Peripartum pulmonary edema is rare, with an incidence of approximately 0.08%; however, it can progress rapidly and cause severe hypoxemia in both the mother and fetus, requiring early diagnosis and appropriate management [1]. In contrast, unilateral pulmonary edema has been reported only in limited clinical settings, and its occurrence during the peripartum period is extremely rare [2]. Consequently, atypical radiographic findings may complicate diagnosis and delay the initiation of appropriate treatment.

During the peripartum period, perioperative management factors, such as the use of tocolytic agents [3,4] and pregnancy-related hemodynamic changes [5,6], have been associated with the development of pulmonary edema. However, their contribution to unilateral pulmonary edema remains unclear. Therefore, we report a case of unilateral pulmonary edema that developed during the management of threatened preterm labor and describe its clinical course, with a focus on the potential role of multiple factors, including body position.

## Case presentation

A 28-year-old woman, gravida 3 para 1, was admitted to our hospital. She was 153 cm tall and weighed 52 kg before pregnancy and 55 kg at admission. She had no history of cardiopulmonary disease, no smoking history, and no notable family history.

At 22 weeks and five days of gestation, she was hospitalized for threatened preterm labor due to cervical shortening. After admission, her vital signs and laboratory parameters were within normal limits, and her peripheral oxygen saturation (SpO₂) was 98%. Oral ritodrine hydrochloride was initiated at a dose of 5 mg four times daily. On hospital day 17 (25 weeks and zero days of gestation), uterine contractions intensified, and treatment was changed to a continuous intravenous infusion of ritodrine hydrochloride at 100 μg/min, with the addition of magnesium sulfate at 2 g/h. Dosages were subsequently adjusted according to the severity of uterine contractions. Throughout the pregnancy, neither hypertension nor proteinuria was observed, and laboratory examinations, including complete blood count and biochemical tests, showed no abnormalities.

The patient strongly believed that pregnant women should maintain a left lateral position and, therefore, spent prolonged periods in a lateral decubitus position. According to her report, she maintained a left lateral decubitus position for approximately 12 hours per day, excluding sleep and toileting, for about two weeks after admission.

**Figure 1:**
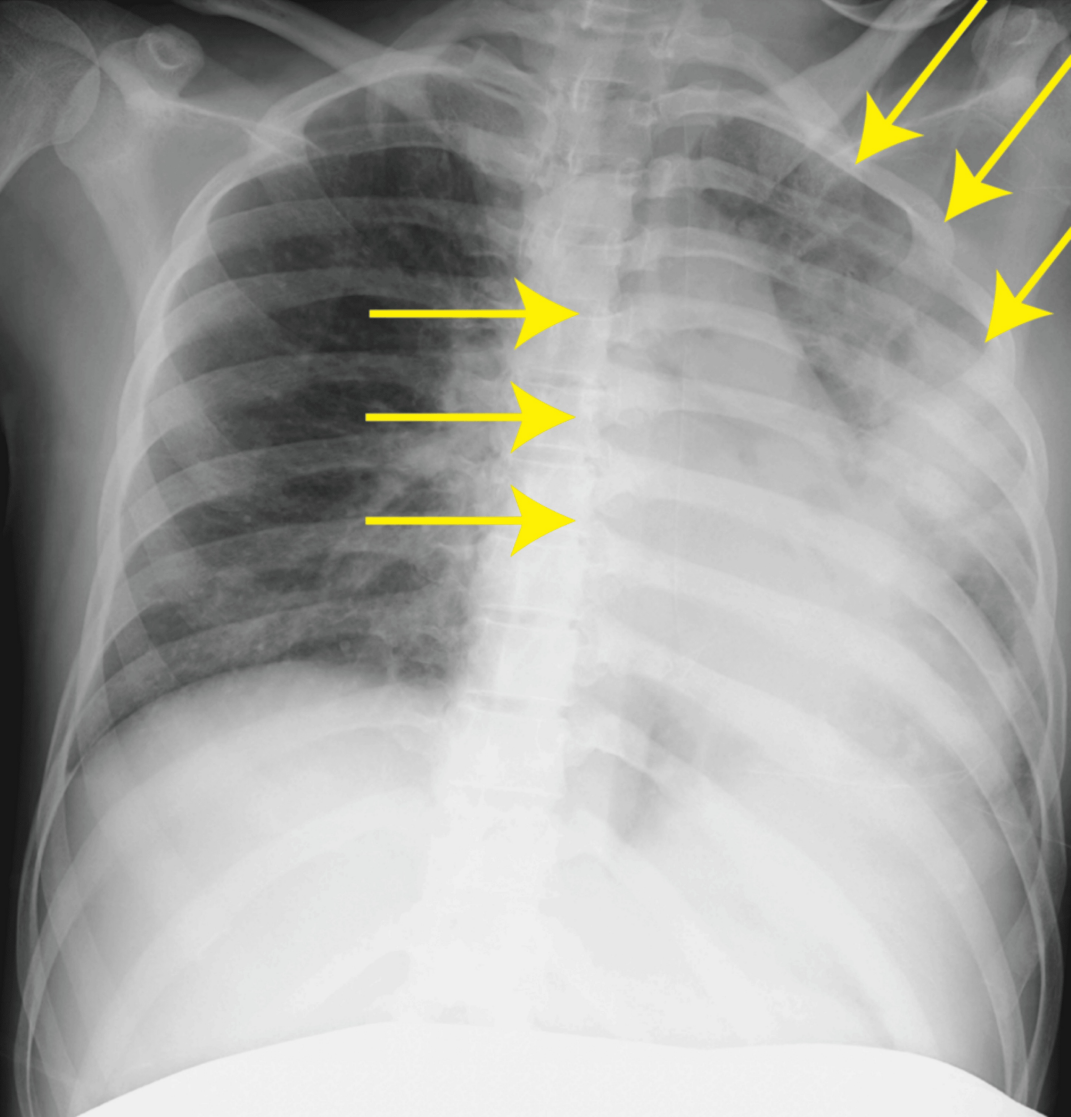
Chest X-ray imaging The area of pulmonary edema is indicated by yellow arrows.

On hospital day 19 (25 weeks and two days of gestation) at approximately 10:00 a.m., she suddenly developed chest pain, palpitations, and dyspnea. Her SpO₂ was 90% on room air. Oxygen therapy was initiated, and a chest X-ray revealed unilateral infiltrative opacities localized to the left lung (Figure 1).

Transthoracic echocardiography showed no evidence of left ventricular wall motion abnormalities, right heart failure, or valvular disease, with no findings suggestive of cardiogenic pulmonary edema.

Considering the possibility of tocolytic agent-associated pulmonary edema, ritodrine hydrochloride and magnesium sulfate were discontinued at 3:00 p.m. on the same day. However, because her respiratory status did not improve, an emergency cesarean section was performed at 5:00 p.m. Spinal anesthesia was administered at the L3/4 interspace using a 27-gauge Quincke needle, with 2.4 mL of 0.5% hyperbaric bupivacaine and 20 μg of fentanyl. Sensory blockade up to the T4 level was confirmed.

As dyspnea persisted after the start of surgery, anesthesia was converted to general anesthesia, induced with propofol, fentanyl, and rocuronium, and maintained with propofol. Invasive arterial blood pressure monitoring was used for perioperative management. Arterial blood gas analysis after tracheal intubation showed a partial pressure of arterial oxygen of 160 mmHg under a fraction of inspired oxygen of 0.6. The surgery was completed uneventfully. The neonate weighed 894 g, with Apgar scores of 5 at one minute and 7 at five minutes. The operative time was one hour and 50 minutes. The total volume of fluids administered preoperatively and intraoperatively was 3,864 mL, urine output was 200 mL, and estimated blood loss was 835 mL.

**Figure 2:**
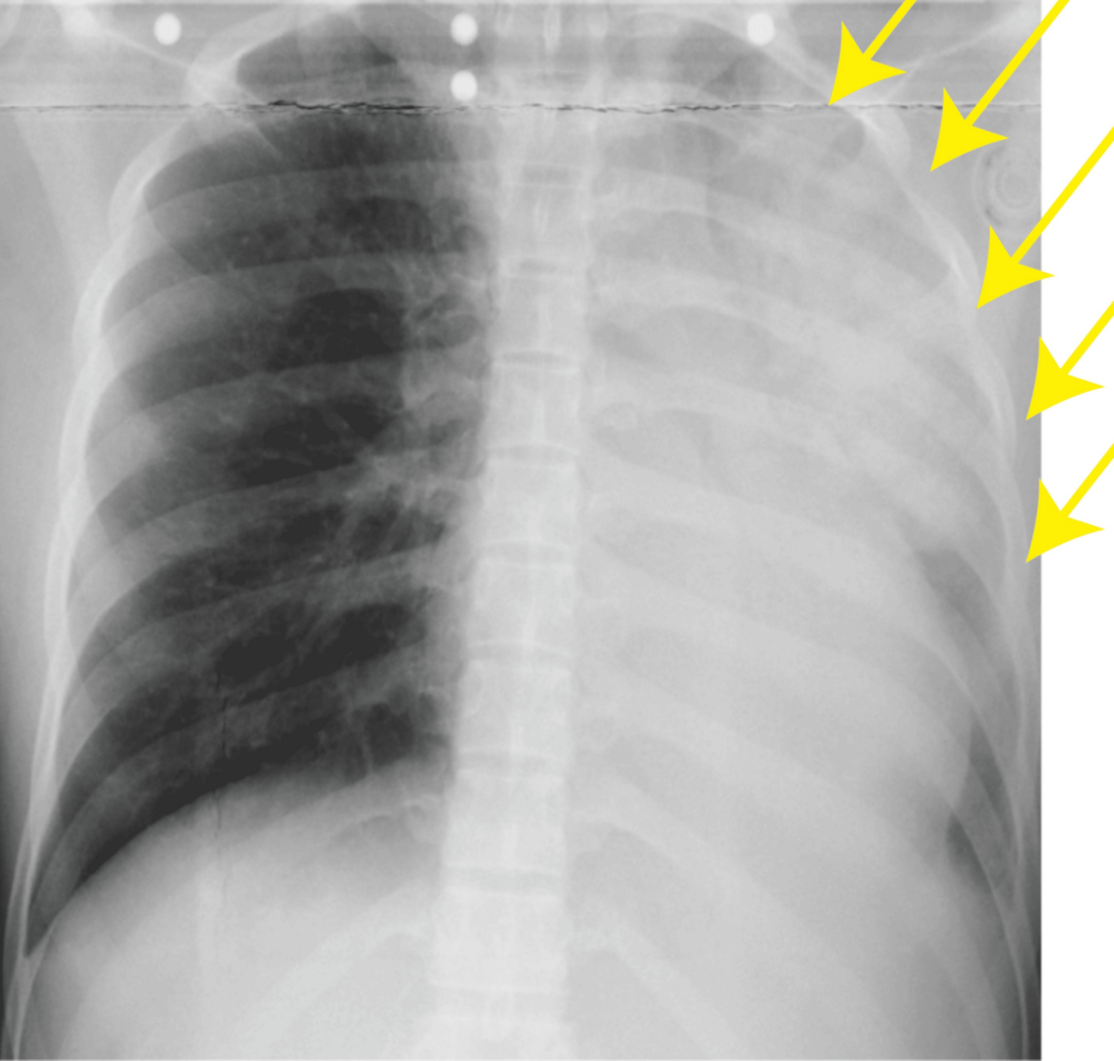
Chest X-ray imaging Postoperative chest X-ray during cesarean delivery. The areas of pulmonary edema are indicated by yellow arrows.

**Figure 3:**
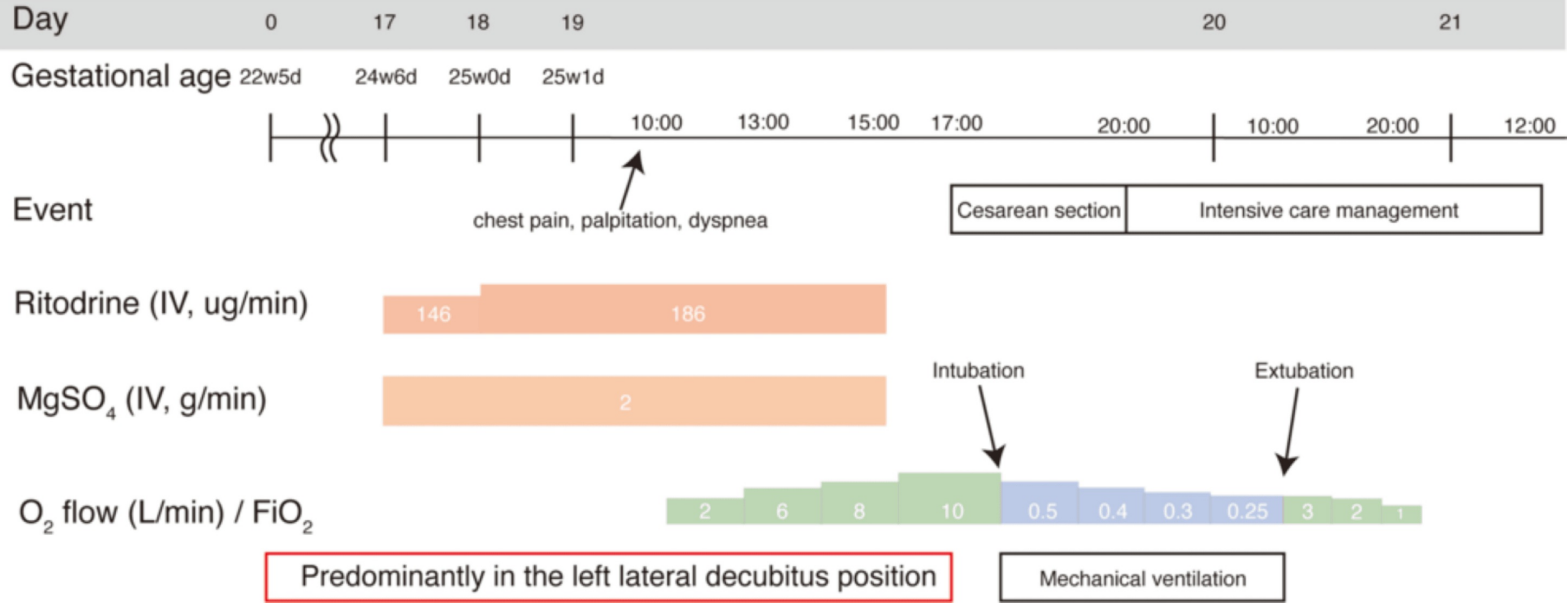
Timeline of clinical course and therapeutic interventions during pregnancy The horizontal axis represents gestational age and hospital days. The figure illustrates changes in tocolytic therapy (ritodrine hydrochloride and magnesium sulfate), respiratory management (oxygen concentration and mechanical ventilation), and major clinical events. At 25 weeks and two days of gestation, the patient developed sudden dyspnea, and a chest X-ray revealed unilateral pulmonary infiltrates in the left lung. Discontinuation of tocolytic agents did not lead to respiratory improvement, and an emergency cesarean section was performed on the same day. Postoperatively, the patient was managed with mechanical ventilation in the intensive care unit, followed by extubation on postoperative day 1 and discontinuation of oxygen therapy on postoperative day 2.

A postoperative chest X-ray demonstrated worsening pulmonary infiltrates (Figure 2), and the patient was admitted to the intensive care unit under continued mechanical ventilation. Her oxygenation gradually improved, and she was extubated on postoperative day 1 after radiographic improvement was confirmed. Oxygen therapy was discontinued on postoperative day 2, and she was discharged from the intensive care unit. She was discharged from the hospital on postoperative day 25 (Figure 3).

## Discussion

Peripartum pulmonary edema may arise from various etiologies, including drug-induced and cardiogenic causes, as well as eclampsia and pregnancy-related hemodynamic changes [1,7]. A case-control study of women with preterm delivery identified spontaneous preterm labor and tocolytic therapy as independent risk factors for peripartum pulmonary edema [7]. In particular, tocolytic agents such as magnesium sulfate and nifedipine have been associated with an increased risk of pulmonary edema [7]. In this case, multiple tocolytic agents were administered to manage threatened preterm labor, consistent with previously reported risk factors, suggesting that peripartum management related to preterm labor may have contributed to the development of pulmonary edema.

Reported causes of unilateral pulmonary edema include mitral regurgitation [8]; rapid re-expansion of the lung after pneumothorax or thoracentesis [9,10]; unilateral pulmonary disorders, such as Macleod syndrome and unilateral absence of the pulmonary artery [11]; compression or obstruction of the pulmonary artery, including aortic aneurysm [12], left ventricular pseudoaneurysm [13], and mediastinal fibrosis [14]; and prolonged maintenance of a unilateral lateral decubitus position [15]. No cardiac dysfunction, structural heart disease, or apparent pulmonary vascular abnormalities were identified in this case. In addition to the use of tocolytic agents for threatened preterm labor, prolonged lateral decubitus positioning before symptom onset may have contributed to the development of unilateral pulmonary edema.

In this case, pulmonary edema developed abruptly, and drug-induced pulmonary edema was suspected based on the clinical course and diagnostic findings. The patient strongly believed that pregnant women should maintain a left lateral position and was confirmed to have spent prolonged periods lying in a lateral decubitus position with the affected side down before surgery. The rapid postoperative clinical and radiographic improvement further suggests that body position, particularly prolonged lateral decubitus positioning, may have contributed to the development of pulmonary edema in this case.

In the lateral decubitus position, gravitational effects increase pulmonary blood flow to the dependent lung, leading to elevated pulmonary capillary hydrostatic pressure [15]. This increase in hydrostatic pressure facilitates fluid transudation from the capillaries into the interstitium and alveoli, contributing to the formation of pulmonary edema. During pregnancy, physiological changes such as increased circulating blood volume and enhanced capillary permeability further predispose patients to pulmonary edema [5]. In addition, lymphatic drainage in the dependent lung may be impaired by gravitational and positional factors, potentially hindering the clearance of interstitial fluid [16]. Sustained pressure loading may also reduce interstitial compliance, exacerbating localized interstitial edema and ultimately resulting in unilateral pulmonary edema. The combined effects of these mechanisms likely led to pulmonary edema localized to the dependent lung, which resolved promptly after positional change and appropriate perioperative management.

This report has several limitations. As a single-case observation, it does not allow definitive conclusions regarding the causal relationship between prolonged lateral decubitus positioning and the development of unilateral pulmonary edema. Although cardiogenic causes were considered unlikely based on clinical evaluation and echocardiographic findings, the potential contributions of tocolytic therapy, pregnancy-related physiological changes, and perioperative fluid management cannot be completely excluded. In addition, detailed hemodynamic or pulmonary perfusion assessments were not performed, which limits further mechanistic interpretation. Nevertheless, the clinical course observed in this case suggests that prolonged maintenance of a lateral decubitus position may be a contributing factor to unilateral pulmonary edema during the peripartum period.

## Conclusions

We report a case of unilateral pulmonary edema occurring during the peripartum period. Because unilateral pulmonary edema is rare and may mimic conditions such as pneumonia, its diagnosis can be challenging. Delayed diagnosis and treatment may lead to serious complications; therefore, early differential diagnosis and prompt management are essential. Anesthesiologists should recognize that prolonged maintenance of a single lateral decubitus position during pregnancy, particularly under tocolytic therapy, may precipitate unilateral pulmonary edema and should be considered during perioperative respiratory assessment.
